# Controlled depolymerisation assessed by analytical ultracentrifugation of low molecular weight chitosan for use in archaeological conservation

**DOI:** 10.1007/s00249-018-1290-6

**Published:** 2018-03-17

**Authors:** Jennifer M. K. Wakefield, Richard B. Gillis, Gary G. Adams, Caitlin M. A. McQueen, Stephen E. Harding

**Affiliations:** 1 National Centre for Macromolecular Hydrodynamics (NCMH), School of Biosciences, University of Nottingham, Sutton Bonington, LE12 5RD UK; 2 School of Chemistry, University of Nottingham, University Park, Nottingham, NG7 2RD UK; 3 School of Health Sciences, University of Nottingham, Queen’s Medical Centre, Nottingham, NG7 2UH UK; 4 Kulturhistorisk Museum, Universitetet i Oslo, St. Olavs plass, Postboks 6762, 0130 Oslo, Norway

**Keywords:** Analytical ultracentrifuge, SEDFIT-MSTAR, Hydrogen peroxide-ultraviolet depolymerisation, Oseberg artefacts

## Abstract

The heterogeneity and molecular weight of a chitosan of low molecular weight (molar mass) and low degree of acetylation (0.1) for potential use as a consolidant for decayed archaeological wood were examined by sedimentation velocity and sedimentation equilibrium in the analytical ultracentrifuge before and after depolymerisation. Sedimentation velocity before depolymerisation revealed a uniform distribution of sedimentation coefficient with little concentration dependence. SEDFIT-MSTAR analysis revealed a weight average molecular weight *M*_w_ of (14.2 ± 1.2) kDa, and polydispersity index of ~ 1.2. Further analysis using MULTISIG revealed a distribution of material between 2 and 20 kDa and consistent with the weight average *M*_w_. Controlled depolymerisation using hydrogen peroxide and ultra-violet radiation in an acetic acid medium reduced this to (4.9 ± 0.7) kDa, with a similar polydispersity. The depolymerised material appears to be within the range that has been predicted to fully penetrate into archaeological wood. The consequences for this finding and the use of the analytical ultracentrifuge in wood conservation strategies are considered.

## Introduction

Chitosan—a polycationic partially deacetylated form of chitin (poly-*N*-acetyl glucosamine)—has been considered for a wide range of applications from fining agents in the fruit juice industry, a condensation agent for DNA therapies, a mucoadhesive, an encapsulant, a film forming agent in shampoos and in wound healing technologies (see for example Harding et al. 2017; Morris et al. 2010, and references cited therein). It also possesses the same backbone—β(1-4) glucan—as cellulose, and this has led to the suggestion of its use as a consolidant to provide strength to archaeological wood where the cellulose has been decayed (Christensen et al. 2015). An example where an appropriate consolidant is needed is in the treatment of excavated artefacts from the Oseberg Viking ship that had been previously treated over 100 years ago with hot alum—potassium aluminum sulfate dodecahydrate—KAl(SO_4_)_2_·12H_2_O (Braovac 2015; Braovac and Kutzke 2012). The crystals formed within the wood on cooling successfully prevented the wooden objects from shrinking upon drying. Unfortunately, this also led to the production of sulfuric acid which over the years has gradually degraded the cellulose (and lignin) making the wood extremely fragile. This has created a need for reconsolidation and there has been a focus on the development of natural polymers for this. Existing consolidants are largely from fossil fuels which could pose a problem in the future. In addition, these can sometimes result in a plastic appearance affecting the way the public view the artefacts. Therefore, more natural consolidants are now being investigated (Christensen et al. 2015; McHale et al. 2016, 2017).

**Fig. 1:**
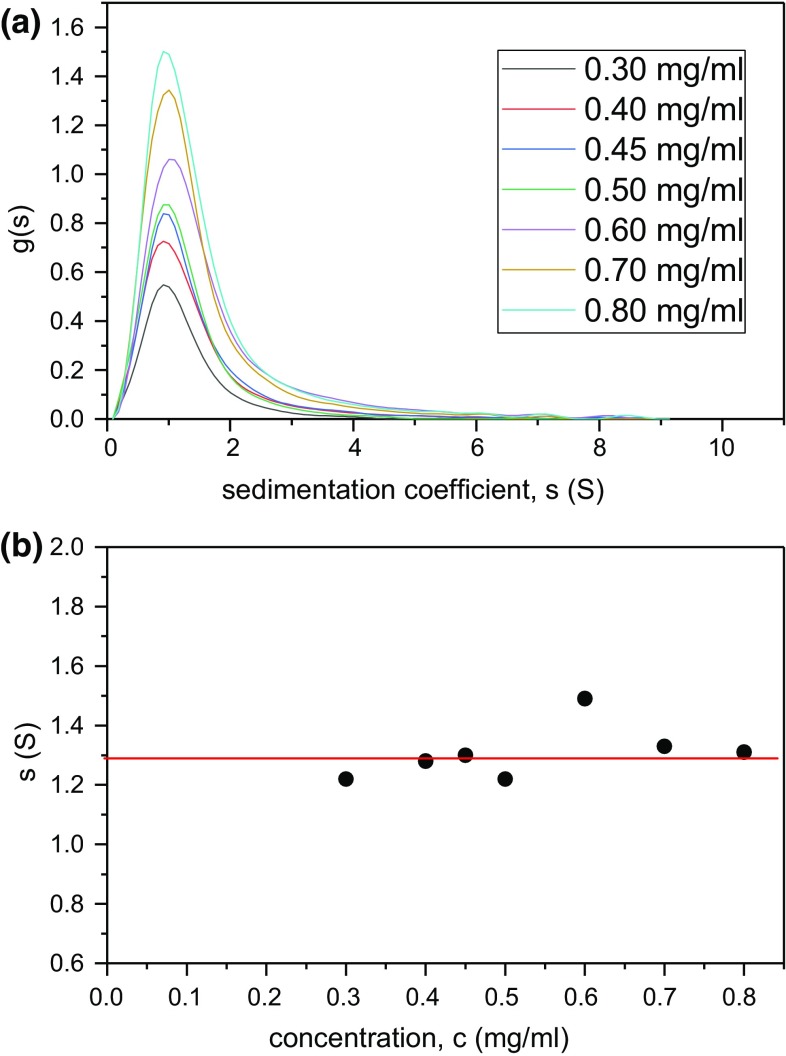
**a** Sedimentation coefficient distribution plots g(*s*) vs* s* (in Svedberg units, S) for Kitonor chitosan at different loading concentrations. **b** Plot of* s* vs concentration,* c* (mg/ml) showing little evidence for significant non-ideality or self-association. The “ideal” value, *s*^o^ = (1.28 ± 0.05) S is taken as the mean, excluding the outlier (> 2 standard deviations away from the mean)

Chitosan (Fig. 1) has already been considered for conservation (Christensen et al. 2015). Besides its similar backbone structure to cellulose, chitosan would appear to have other potentially favourable properties, namely absorption of protons (lowering the effect of acid), chelation of metal ions, antifungal activity, ease of functionalisation as well as the potential for interaction with lignin. Christensen et al. (2015) examined a chitosan preparation with a molecular weight (estimated by gel permeation chromatography, GPC, relative to pullulan standards) of ~ 12 kDa. Penetration experiments showed that some of that chitosan could be introduced into the wood fibre, but uptake was improved when the molecular weight was reduced to ~ 6 kDa. Therefore, it was considered that future consolidants based on chitosan should be depolymerised to this level. The depolymerisation needs to be controlled as the material has to be small enough to be taken up but not so small as to require the chitosan to be extensively re-polymerised inside the wood to provide the appropriate strength. Uncertainty remains from the previous column chromatography study (Christensen et al. 2015) about the molecular weights due to the different conformation of the standard used (pullulan—a random coil) with chitosan (a much stiffer polymer) and due to possible interactions with the column. Analytical ultracentrifugation, on the other hand, is an absolute (no standard required) and matrix-free alternative, and these features, together with its inherent separation ability, make it the method of choice.

Molecular weights of polysaccharides can be reduced through various means (Harding 2010), such as the application of treatment with hydrogen peroxide with thermal processing, microwaves, UV light or potassium nitrite (Chang et al. 2001; Christensen et al. 2015; Ma et al. 2014; Mao et al. 2004; Morris et al. 2009a; Tian et al. 2003; Wang et al. 2005). For conservation, the target molecular weights for the depolymerised material are generally ~ < 5 kDa, considerably lower compared with those obtained from natural sources (shells of crustaceans or some mushroom species). As a comparison, polyethylene glycol (PEG) preparations also used in conservation are generally between 0.2 and 4 kDa depending on the extent of degradation of the wood being treated (Jenssen 1987).

For the present study, we focussed on “Kitonor” (Norwegian Chitosan, Gardermoen, Norway) used by Christensen et al. (2015) and used the analytical ultracentrifuge as our method of enquiry. Developments and use for polysaccharide characterisation have been recently reviewed (Harding et al. 2015) and the method has been well established for the characterisation of chitosan and chitosan nanoparticles (Almutairi et al. 2015; Cölfen et al. 1996; Fiebrig et al. 1994; Harding et al. 2015; Morris et al. 2009a, b, 2011). We first of all characterized its heterogeneity using sedimentation velocity in the analytical ultracentrifuge which gives the sedimentation coefficient distribution. We then accurately measured its weight average molecular weight by using the absolute (i.e., not requiring calibration standards of assumed similar conformation) technique of sedimentation equilibrium and analysed using the SEDFIT-MSTAR procedure of Schuck et al. (2014). This was followed by establishing the molecular weight distribution using MULTISIG (Gillis et al. 2013), taking advantage of the low molecular weight and hence low thermodynamic non-ideality (confirmed by sedimentation velocity). The Kitonor chitosan was then depolymerised by treatment with hydrogen peroxide and ultra-violet (UV) radiation in an acetic acid medium. The depolymerisation process was also repeated 5 times to ensure the results were consistent. Finally, depolymerisation over time was also investigated to show that the degree of polymerisation can be controlled by the reaction time, as monitored by the weight average molecular weights (*M*_w_). The consequence of this for application to alum treated wood was then considered.

## Materials and methods

Kitonor chitosan was used (Christensen et al. 2015). The chitosan comes from crab with a degree of acetylation DA = 0.1 (with < 2% ash content) supplied by Norwegian Chitosan (Gardermoen) Ltd. Samples were dissolved in an acetate buffer, comprising 0.2 M acetic acid and 0.2 M sodium acetate, pH = 4.3.

### Depolymerisation of chitosans

Degradation of chitosan largely followed the procedure of Wang et al. (2005). 4% chitosan was dissolved, with stirring in 0.2% acetic acid for 1 h. After obtaining a clear solution, 4% hydrogen peroxide was added to obtain a 2% chitosan in a buffer containing 0.1% acetic acid and 0.2% hydrogen peroxide. This preparation was exposed to UV light (wavelength 254 nm at 0.04 mW.cm^-2^) for 1 h at 20 °C. For UV exposure, the volume to surface area ratio was kept constant. The solution was then neutralized with 1 M sodium hydroxide. The precipitate was then centrifuged (6 × 60 ml vials) at 10,000 revolutions per minute (rpm) and rinsed 3 times with 50 ml deionised H_2_O per vial. The solid product was frozen in a − 80.0 °C freezer overnight and freeze dried, resulting in a yield of 66–78%. The reaction was also carried out for 30 min and 90 min to investigate the effect of reaction time on *M*_w_.

### Sedimentation velocity in the analytical ultracentrifuge

A Beckman XL-I analytical ultracentrifuge (AUC) was used and equipped with Rayleigh interference optics. 12 mm optical path double sector cells were employed: solution and solvent (buffer) reference channels were filled to 400 μl. A rotor speed of 50,000 rpm was used at a temperature of 20.0 °C. A 1.0 mg/ml stock chitosan solution in 0.2 M acetate buffer stock solution was prepared as above and then diluted to 0.80, 0.70, 0.60, 0.50, 0.45, 0.40 and 0.30 mg/ml. Analysis was carried out using SEDFIT (Dam and Schuck 2004) which gave distributions of sedimentation coefficient g(s) vs s, and the corresponding weight average sedimentation coefficient. All sedimentation coefficients were normalized to standard conditions (density and viscosity of water at 20.0 °C)—see Tanford (1961). A value for the partial specific volume *ῡ* = 0.57 ml/g was used (see Errington et al. 1993).

### Sedimentation equilibrium in the analytical ultracentrifuge

The Beckman XL-I AUC was also used as above. For experiments on the native, undepolymerised Kitonor chitosan a 20 mm path length cell was used, with short (145 µl) columns and a rotor speed of 35,000 rpm at a temperature of 20.0 °C. For the depolymerisation experiments, a higher rotor speed of 40,000 rpm (with 12 mm cells, 100 µl) was employed. Analysis was carried out using SEDFIT-MSTAR (Schuck et al. 2014) which provides the (apparent) weight average molecular weight *M*_w,app_ (obtained from both M* analysis of Creeth and Harding 1982, and the hinge point method—see Schuck et al. 2014). Loading concentrations from 0.40 to 1.0 mg/ml were employed to monitor for any associative or non-ideal effects and these were negligible. For the native chitosan, as a further check additional runs were performed at 40,000 and 48,000 rpm to monitor for any speed dependence effects. MULTISIG (Gillis et al. 2013)—which assumed thermodynamic ideality—was also run using a 17 component system with 20 iterations for each concentration, to obtain molecular weight distributions.

## Results and discussion

### Heterogeneity of Kitonor chitosan by sedimentation velocity

Figure 1a shows the sedimentation coefficient distribution, g(s) vs s of the (untreated) Kitonor. For all concentrations, the plots appear unimodal with log-normal polydispersity, with very little evidence of concentration dependence (Table 1 and Fig. 1b) as compared with larger carbohydrate systems. This indicates that for these low molecular weight polysaccharides in the presence of supporting electrolyte, non-ideality is not significant.

**Table 1:** Comparison of* s* (from SEDFIT) and *M*_w,app_ (from SEDFIT-MSTAR) values for untreated Kitonor chitosan. *s*^o^ = (1.28 ± 0.05) S, *M*_*w*_ = (14.2 ± 1.2) kDa (from 35,000 rpm data)

c (g/ml)	*s* (S)	^a^*M*_w,app_ (kDa)	^b^*M*_w,app_ (kDa)	^c^*M*_w,app_ (kDa)
0.3	1.3	15.7	–	10.6
0.4	1.3	12.9	11.9	–
0.45	1.3	–	–	–
0.5	1.2	14.8	12.5	10.3
0.6	1.5	14.8	14.9	–
0.7	1.3	12.3	13.2	11.4
0.8	1.3	15.2	14.3	–
1.0	–	14.0	12.3	–

^a^35,000 rpm

^b^40,000 rpm

^c^48,000 rpm

Evaluations were not always possible at the higher speeds

No sedimentation velocity was performed at 1 mg/ml, no sedimentation equilibrium at 0.45 mg/ml (7 hole rotor limit)

## Weight average molecular weight and molecular weight distribution of untreated Kitonor chitosan

Table 1 compares the apparent weight average molecular weights from SEDFIT-MSTAR as a function of concentration (Schuck et al. 2014) for the three rotor speeds used, with the weight average apparent molecular weights *M*_w,app_ obtained using the hinge point method. Because of the high polydispersity of the samples this method was more reliable than the M* extrapolation for the untreated materials (which involves an extrapolation of the M* function to the cell base).

**Fig. 2:**
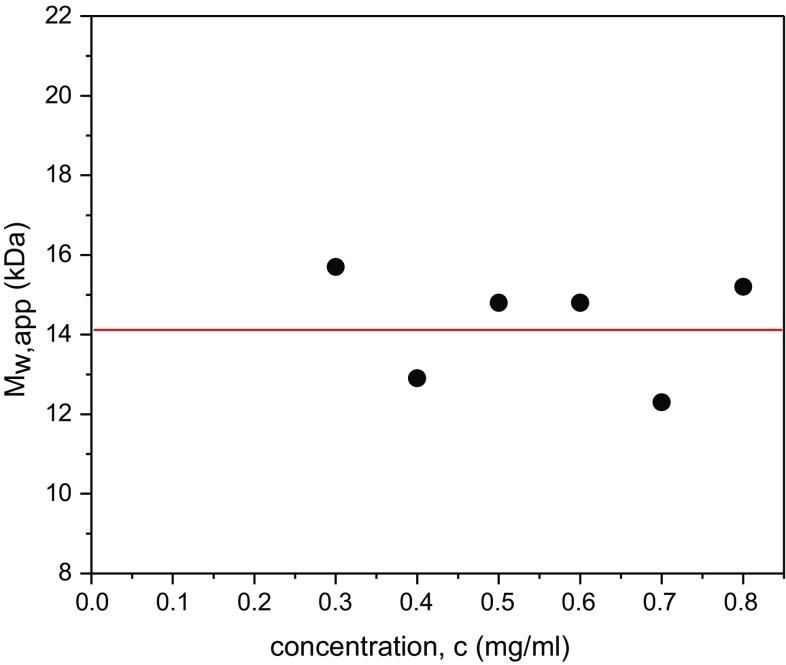
Plot of apparent weight average molecular weight *M*_w,app_ vs loading concentration, c for untreated Kitonor chitosan. SEDFIT-MSTAR used with the hinge point method to extract *M*_w,app_ values. Rotor speed = 35,000 rpm. The ideal value *M*_w_ = (14.2 ± 1.2) kDa

The molecular weights determined with the two lowest speeds (35,000 and 40,000 rpm) gave comparable data but at the highest speed (48,000 rpm) a significant reduction was found, a consequence of the loss from optical registration at the cell base of some the higher molecular weight part of the (polydisperse) distribution. This demonstrates that the popular practice used for protein work of using global analysis methods which average results over different speeds is unsuitable for highly polydisperse polysaccharides such as those being studied here. No significant change in molecular weight with concentration is observed (Fig. 2), indicating that non-ideality effects are small, so it is reasonable to assume that the ideal value *M*_w_ ~ the mean *M*_w,app_ without the need for an extrapolation to *c* = 0. *M*_w_ = (14.2 ± 1.2) kDa.

**Fig. 3:**
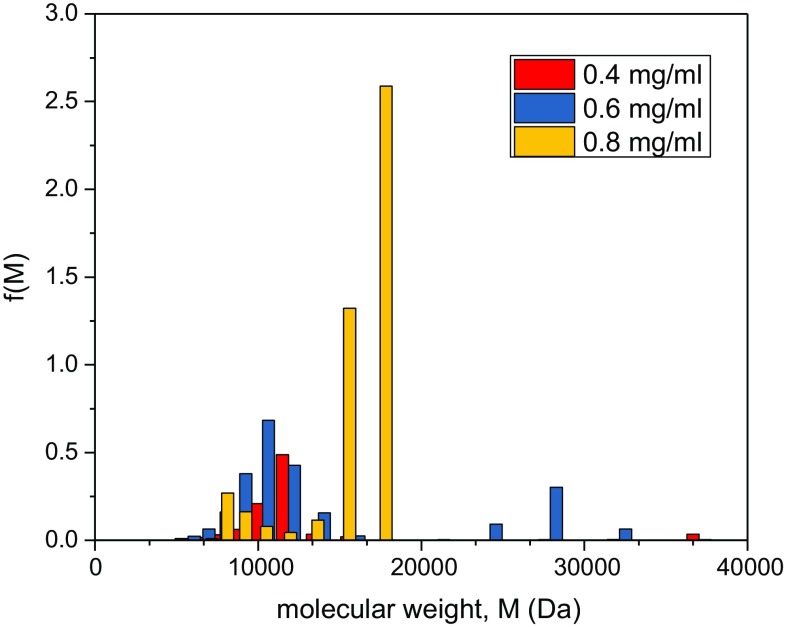
MULTISIG analysis of the molecular weight distribution f(*M*) vs* M* of Kitonor chitosan run at 35,000 rpm at three concentrations. *M*_w_ = (14.1 ± 1.2) kDa, *M*_z_ = (16.4 ± 1.2) kDa with a polydispersity index *M*_z_/*M*_w_ ~ 1.2

To estimate the distribution of molecular weights f(*M*) vs* M*, MULTISIG analysis (Gillis et al. 2013) was run on three concentrations (Fig. 3), where the polydisperse distribution is approximated as an ideal 17-component system. As can be seen from Figs. 1 and 2, the assumption of thermodynamic ideality is a reasonable one. MULTISIG analysis revealed a distribution ranging between 5 and 37 kDa with components peaking between 10 and 17 kDa with an overall weight average of *M*_w_ ~ (14.1 ± 1.2) kDa in exact agreement with SEDFIT-MSTAR. The analysis also yields an *M*_z_ = (16.4 ± 1.2) kDa giving a polydispersity *M*_z_/*M*_w_ ~ 1.2.

Molecular weights > 10 kDa might not be able to penetrate the wood. By comparison, the polyethylene glycol PEG, popularly used in conservation, at present has a molecular weight of 4 kDa or less. Depolymerisation was, therefore, carried out as described in the methods in an attempt to bring the size of the chitosan down to a comparable level.

### Depolymerisation

**Fig. 4:**
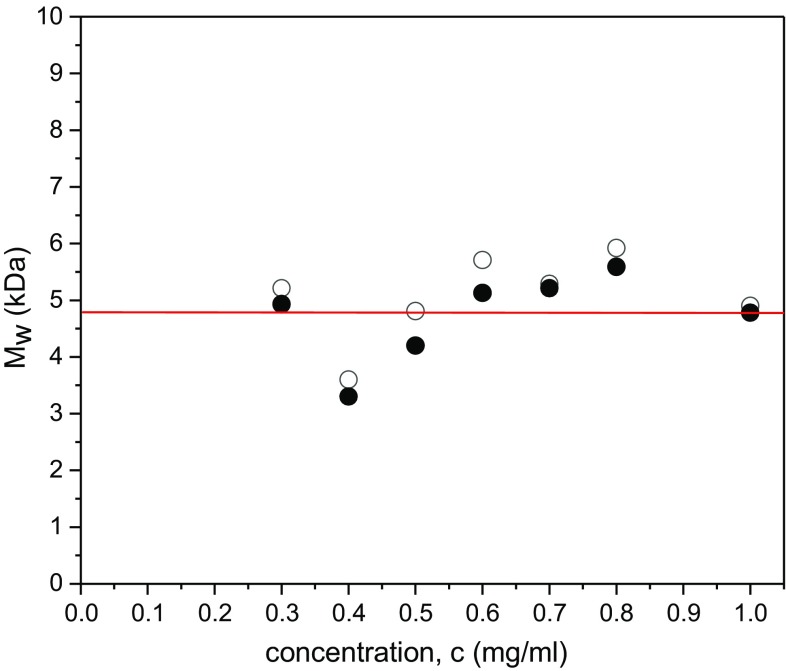
Plot of *M*_w,app_ from SEDFIT-MSTAR vs loading concentration, c for depolymerised chitosan (treated for 60 min) run at 40,000 rpm. Non-ideality is negligible over the concentration range studied with *M*_w_ ~ *M*_w,app_ = (4.9 ± 0.7) kDa. Filled circles: from hinge point method. Open circles from extrapolation of M* method

The effects of depolymerisation treatment for 60 min on the molecular weight of the Kitonor chitosan are shown in Fig. 4: this shows *M*_w,app_ values estimated from SEDFIT-MSTAR analysis of the sedimentation equilibrium records plotted versus loading concentration* c*. Following the analysis procedure for Fig. 2 (untreated), we obtain for the depolymerised material a value of *M*_w_ = (4.9 ± 0.7) kDa, a clear reduction in molecular weight compared with the native material of ~ 60%.

**Fig. 5:**
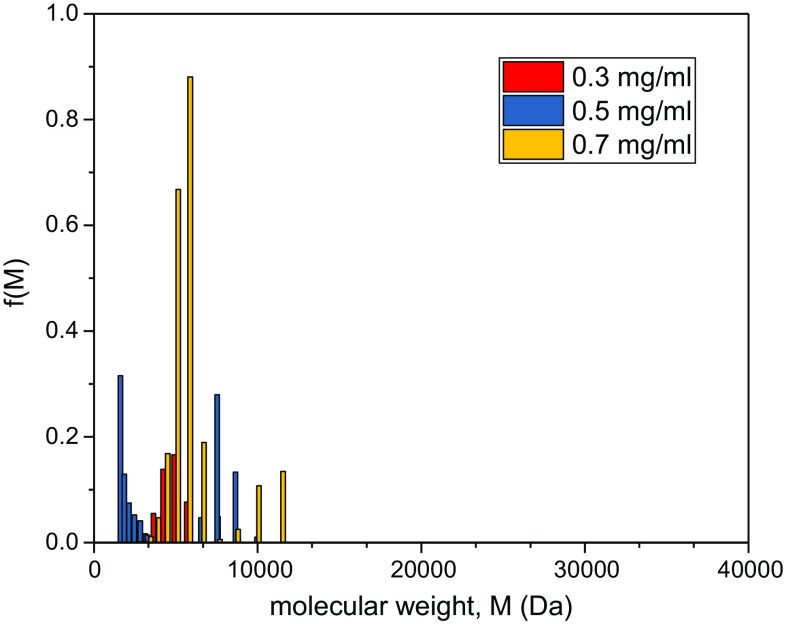
MULTISIG analysis of the molecular weight distribution f(M) vs M of depolymerised (treatment for 60 min) Kitonor chitosan run at 35,000 rpm at three concentrations. *M*_w_ = (5.2 ± 0.7) kDa, *M*_z_ = (6.1 ± 0.5) kDa, and *M*_z_/*M*_w_ ~ 1.2. A clear shift to low molecular weights compared with Fig. 3 is seen

Although sedimentation velocity was not possible for assessing heterogeneity due to the small sizes, MULTISIG analysis of the sedimentation equilibrium data was still possible. Figure 5 shows a significantly reduced distribution of molecular weight, and as a result the majority of the material will be in the molecular weight range most likely to penetrate the archaeological wood. M_w_ from MULTISIG = (5.2 ± 0.7) kDa, again in good agreement with SEDFIT-MSTAR, and much lower than the untreated sample. From MULTISIG, we also obtained an estimate for the z-average, *M*_z_ = (6.1 ± 0.5) kDa, giving a polydispersity *M*_z_/*M*_w_ ~ 1.2, similar to the chitosan prior to depolymerisation, consistent with the higher molecular weights being depolymerised first (see Mao et al. 2004).

**Fig. 6:**
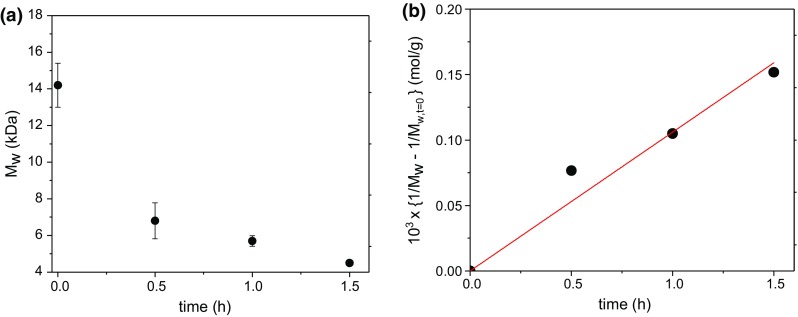
Degradation of Kitonor chitosan as a function of treatment time with hydrogen peroxide and UV radiation. **a** Reduction of weight average molecular weight. The error bars represent the average over different concentrations. **b** Corresponding plot of {1/*M*_*w*_ − 1/*M*_*w,t*=0_} vs time (Tanford 1961). Decay constant *k* = (0.046 ± 0.004) h^−1^

To determine the effect of reaction time and investigate how the molecular weight of chitosan can be controlled, treatments at 0.5, 1.0 and 1.5 h for each of 3 concentrations (0.5, 0.6 and 0.7 mg/ml) were investigated. The results from this are shown in Fig. 6.

Treatment with hydrogen peroxide coupled with UV light appears to degrade the chitosan in a controlled way. 30, 60 and 90 min reaction times show that the degree of depolymerisation can be appropriately chosen, at least for Kitonor chitosan. What is apparent from Fig. 6a is that there is an initial large decrease in molecular weight which tails off exponentially. A dramatic decrease in molecular weight in the first 30 min is also observed for depolymerisation of much larger molecular weights with hydrogen peroxide and UV light and also hydrogen peroxide alone (Ma et al. 2012; Wang et al. 2005).

We can estimate a depolymerisation decay constant k from the relation of Tanford (Eq. 33.12 of Tanford 1961) which provides a good approximation to the initial stages of the decay process—see also Holme et al. (2008) and Morris et al. (2009a):

1 $$ \{{ 1/x_w {-} 1/x_{w, \, t = 0} }\} = (k/2) \cdot t $$

Since the weighted average degree of polymerisation *x*_*w*_ is just (*M*_*w*_/*m*_o_) with *m*_o_ the molecular weight of the repeat unit, Eq (1) is just

2 $$ \{{ 1/M_w {-} 1/M_{w, \, t = 0} }\} = (k/ 2m_{\text{o}} ) \cdot t $$

with *m*_o_ for chitosan = 216 Da. From the slope of Fig. 6b, this leads to an estimate for *k* ~ (0.046 ± 0.004) h^−1^.

### Scale-up

The effect of “scale-up”—processing of larger quantities of material—was then investigated. 5 × 22 g batches of chitosan were each depolymerised for 1 h. Encouragingly these consistently gave the same molecular weight *M*_*w*_ = (5.35 ± 0.70) kDa (three concentrations were used 0.5, 0.6 and 0.7 mg/ml for each batch) which proves the consistency of the results. Reproducibility can also be an issue with depolymerisation. However, keeping the same concentrations, reaction times and volume to surface area ratio, it is clearly possible to get similar molecular weights using the same starting material.

To increase the quantity of chitosan being depolymerised, an in-flow UV depolymerisation system could be set up but the reaction times may need to be adjusted to fit with the change in surface area exposure to UV light and volume.

### Concluding remarks

In this study, we have demonstrated that it is possible to produce chitosan of a suitable molecular weight range—which could form the basis for a consolidant for archaeological wood and in a controllable fashion. We have also established a procedure for determining the molecular weights and molecular weight ranges of consolidant materials on an absolute and matrix-free basis and for determining their stability against decay.

We have investigated chitosan at one degree of acetylation DA = 0.1. Examination of chitosans of other DA’s may prove useful, although the stability of chitosans seems to be independent of DA (Holme et al. 2008).

This paper was presented at 5th EPNOE International polysaccharide conference held in Jena, Germany, August 2017 and at the 23rd analytical ultracentrifugation conference held in Glasgow, UK, September 2017.
